# The Role of Long Non Coding RNAs in the Repair of DNA Double Strand Breaks

**Published:** 2017-01-17

**Authors:** Ali Dianatpour, Soudeh Ghafouri-Fard

**Affiliations:** *Department of Medical Genetics, School of Medicine, Shahid Beheshti University of Medical Sciences, Tehran, Iran* *.*

**Keywords:** DNA damage response, long non coding RNA, non- homologous end-joining (NHEJ), homologous recombination (HR)

## Abstract

DNA double strand breaks (DSBs) are abrasions caused in both strands of the DNA duplex following exposure to both exogenous and endogenous conditions. Such abrasions have deleterious effect in cells leading to genome rearrangements and cell death. A number of repair systems including homologous recombination (HR) and non-homologous end-joining (NHEJ) have been evolved to minimize the fatal effects of these lesions in cell. The role of protein coding genes in regulation of these pathways has been assessed previously. However, a number of recent studies have focused on evaluation of non-coding RNAs participation in DNA repair. We performed a computerized search of the Medline/ Pubmed databases with key words: DNA repair, homologous recombination, non-homologues end joining and long non-coding RNA (LncRNA). The existing data highlight the role of long non-coding RNAs in DSB repair as well as dysregulation in their expression which would lead to pathological conditions such as cancer. The specific mechanism of their contribution in DNA repair pathways has been elucidated for a few of them. LncRNAs participate in several steps of DNA repair pathways and regulate the expression of key components of these pathways including p53 tumor suppressor gene.

DNA double-strand breaks (DSBs) are abrasions caused in both strands of the DNA duplex as the result of exposure to either exogenous or endogenous conditions. Ionizing radiation (IR) is the most important exogenous agent causing DSB, while endogenous source happens when DNA replication forks run into unrepaired DNA abrasions, inducing fork collapse. Irrespective of their cause, DSBs have deleterious effect in cells leading to genome rearrangements and cell death (1). Stress response pathways which are induced after intrinsic and extrinsic DNA damages are called DNA damage response (DDR). Collectively, these pathways protect the genome and the continued existence of cells (2). DSBs as the most fatal DNA abrasions are sensed by a principal kinase named ataxia-telangiectasia mutated (ATM). Following DNA damage, ATM is activated by autophosphorylation at damage regions and sequentially phosphorylates a variety of downs-tream substrates such as the tumor protein 53 (TP53), breast cancer type 1 susceptibility protein (BRCA1) and checkpoint kinase 2 (CHK2). The DNA damage signal which is relayed by these effectors induces cell-cycle checkpoints, DNA repair, and apoptosis (3). A number of DSB repair mechanisms have been developed in various organisms to prevent genotoxic effects (1). Alternatively, DSBs are scheduled by the cell in some conditions such as during the first meiotic division which participate in the homologous recombination and chromosome pairing during prophase I (4) as well as generation of rearrangements at immunoglobulin genes which are crucial for the diversity of antigen receptors produced from restricted loci (5). Failures in DSB repair system have led to a number of developmental, immunological, and neurological diseases and more prominently are involved in the tumorigenesis process (6). The two main evolutionary conserved DSB repair pathways are homologous recombination (HR) and non-homologous end-joining (NHEJ). NHEJ refers to the pathway in which the break ends are straightly ligated without the requisite for a homologous template which makes the process error-prone, while in the HR a homologous sequence is required to direct the repair. In NHEJ, the ATP-dependent DNA helicase 2 subunit Ku70/80-like proteins (Ku70-Ku80) heterodimer (Ku) hold the DSB ends in close vicinity to enhance their direct ligation. The clamp-like complex is arranged and then DNA-dependent protein kinases, catalytic subunits (DNA-PKcs) are recruited to the damaged site. A number of other proteins, such as Artemis, DNA ligase IV, X-Ray repair cross complementing 4 (XRCC4) and XRCC4-like factor (XLF), gather with the Ku80, Ku70 and DNA-dependent protein kinase (DNA-PK) complex and participate in the DNA repair pathway (7). NHEJ is an important pathway of DSB repair in mammalian cells as well as a pathway implicated in the construction of antigen receptor genes and telomere preservation (8, 9). On the other hand, HR is a more precise repair mechanism recruited when the DSB is excised by nucleases and helicases, making 3' single-stranded DNA (ssDNA) overhangs onto which the RAD51 recombinase accumulates. This construction can attack homologous duplex DNA to employ it as a template for DNA repair. NHEJ participates in DSB repair all over the cell cycle but more prominently in G1 cells, whereas HR is more active after DNA replication when an identical sister chromatid exists as a template for repair (1). Tens of regulatory proteins have been identified that determine the choice of pathways in each cell cycle phase or regulate each repair pathway (1).

Recently, many studies have focused on a group of mRNA-like transcripts that do not code for proteins and have other distinctive features when compared to mRNAs. Such transcripts have the length of more than 200 nucleotides which make them distinct from classic non coding RNAs such as ribosomal (r)RNAs, ribozymes, transfer (t)RNAs and microRNAs (10). These long non coding RNAs (lncRNAs) are major participants in many fundamental biologic processes including regulation of gene expression, telomere length, chromatin rearrangement, histone modification, modification of alternative splicing, dosage compensation, genomic imprinting and cell differentiation (11-14). According to GENCODE annotations human genome consists of more than 15,900 lncRNA genes (15). The first reports regarding the role of lncRNAs in DDR was the detection of transcripts produced upstream of the cyclin D1* (CCND1)* promoter following DNA damage which subsequently bind to an RNA-binding protein participating in DNA repair, leading to inhibition of histone acetyl transferase CREB-binding protein (CBP)/p300 and down-regulation of *CCND1*, a cell cycle regulator (16). Recent studies have shown involvement of numerous ncRNAs in several stages of DSB repair. However, most of studies on ncRNAs implicated in the DDR have up to now concentrated on the role of miRNAs (17). Consequently, in the present study, we reviewed the existing data regarding the role of lncRNAs in DSB repair as well as any dysregulation in their expression which would lead to pathological conditions such as cancer.

**Evidence acquisition**

We implemented a computerized search of the Medline/ Pubmed, Web of Knowledge, Scopus, ProQuest and Google Scholar databases with key words: DNA repair, homologous recombination, non-homologues end joining and long non-coding RNA within the maximal date range until 2016.

**LncRNAs role in DNA repair pathways**

Numerous lncRNAs have been shown to participate in DNA repair pathways. Figure 1 shows the position of selected lncRNAs in the mentioned pathways. Additional information about their chromosomal location and function is provided in table 1. For the reason of simplification, lncRNAs have been categorized based on their participation in HR or NHEJ.

**LncRNAs role in homologous recombination**

***Antisense noncoding RNA in the INK4 locus (ANRIL)***

This lncRNA is encoded from a hotspot region for disease- associated polymorphisms on chromosome 9p21. The polymorphisms in this chromosomal region have been shown to be associated with a wide range of human diseases including cancer. *ANRIL* has a regulatory effect on its adjacent tumor suppressors cyclin dependent kinase inhibitor 2A/B* (CDKN2A/B)* by epigenetic mechanisms and by this means control cell proliferation and senescence (18). *ANRIL *partic-ipates in the preservation of DDR through controlling the cell cycle checkpoints, apoptosis and DNA repair. Its transcription has been demonstrated to be enhanced by the E2F transcription factor 1 (E2F1) in an ATM- dependent manner after DNA damage. Increased levels of *ANRIL* inhibit the expression of *CDKN2A/B* and alternate reading frame (*ARF*) at the late-stage of DDR. Such expression change permits the cell to come back to the normal conditions when DNA repair has finished. In brief, *ANRIL* hinders cell cycle checkpoints and enhances cell cycle progression in the DDR. HR pathway has been shown to be significantly reduced in *ANRIL*-depleted cells, implying that *ANRIL* is essential for the effectiveness of this DNA repair system (19).

**Fig. 1:**
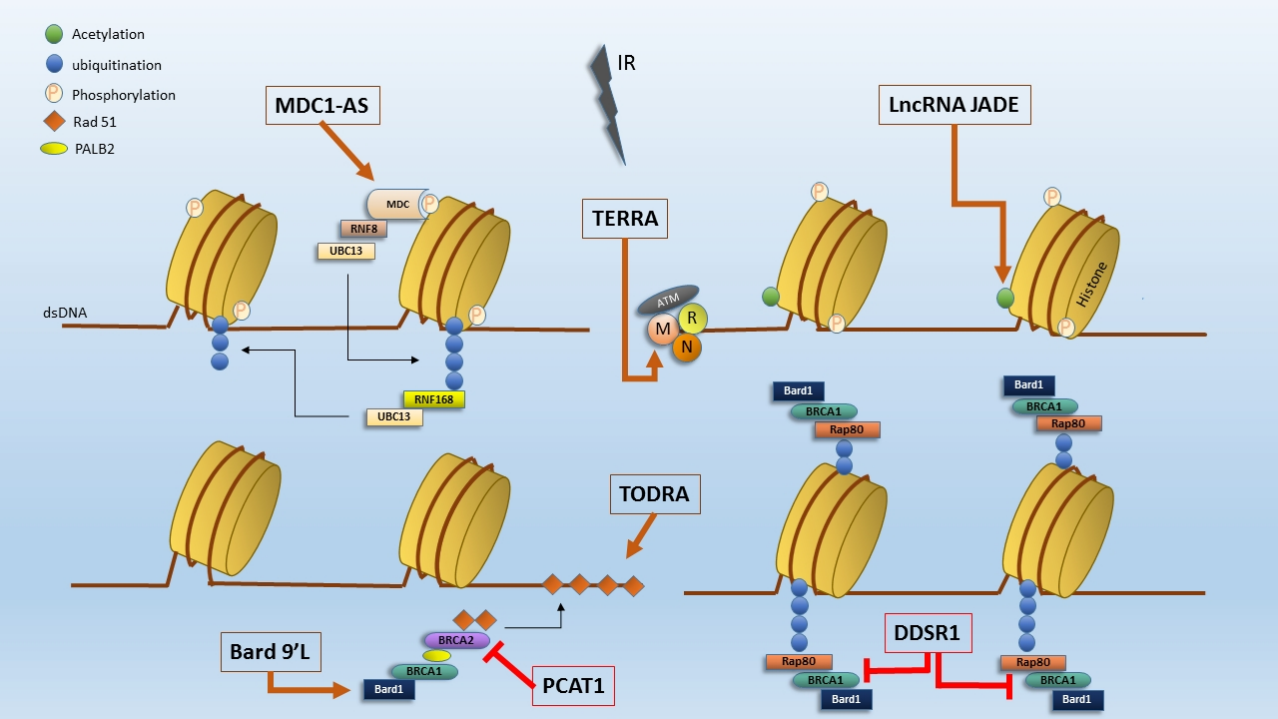
Several lncRNAs are involved in different steps of DNA damage response. IR: ionizing radiation, BARD1 9'L: BRCA1-associated RING domain protein 1 9'L, PCAT1: prostate cancer associated transcript 1, MDC-AS1: mediator of DNA damage checkpoint 1- antisense RNA, TERRA: telomeric repeat-containing RNA, TODRA: transcribed in the opposite direction of RAD51, LncRNA-JA DE: LncRNA-Jade family plant homeo domain (PHD) finger, DDSR1: DNA damage-sensitive RNA 1, dsDNA: double stranded DNA, MDC: mediator of DNA damage checkpoint 1, RNF8: ring finger protein 8, UBC13: E2 ubiquitin-conjugating protein UBC13, RNF168: ring finger protein 168, BRCA1: breast cancer 1, DNA repair associated, BRCA2: breast cancer 2, DNA repair associated, MRN complex: Mre11, Rad50, Nbs1, ATM: ataxia telangiectasia mutated, Rap80: ubiquitin interaction motif containing 1(UIMC1), PALB2: partner and localizer of BRCA2, Rad51: RAD51 recombinase (RAS associated with diabetes protein 51).

**Table 1:** LncRNAs involved in DNA repair pathways and their role in tumorigenesis

**LncRNA**	**Chromosome location**	**Role in tumorigenesis**	**Targets**	**Function**	**DNA repair pathway**	**References**
*DDSR1*	12q23.3	Tumor suppressor	*BRCA1* *hnRNPUL1*	Fine-tunes the recruitment of BRCA1 to DSBs upon DNA damage	HR	23
*LncRNA-JADE*	4q28.2	Oncogene	*JADE* *BRCA1*	Activates the expression of JADE1 and induces histone H4 acetylation in the DDR	HR	3
*TODRA*	15q15.1	Tumor suppressor	*TPIP*	Increase transcription of Rad51 through the expression induction of TPIP	HR	31
*MDC1-AS*	6p21.33	Tumor suppressor	*MDC1*	Plays tumor suppressor role through up-regulation of its antisense tumor-suppressing gene MDC1	HR	28
*PCAT1*	8q24.21	Oncogene	*BRCA2* *PT*	Post transcriptional repression of the BRCA2	HR	30
*ANRIL*	9p21.3	-	*P14 ARF* *P15 INK4b* *P16 INK4a*	Suppresses the expression of INK4a, INK4b and ARF	HR	18
*TERRA*	Subtelomeric	-	*MRE11* *LSD1* *Ku70 Ku80*	Facilitates LSD1-MRE11 interaction; LSD1 enhances the nuclease activity of MRE11 in vitro	HR, NHEJ	44-47
*LINP1*	Chr 10	-	*DNA-PKs* *Ku70-Ku80*	Provides a scaffold for Ku80 and DNA-PKcs	NHEJ	32
*Wrap53* *α*	17p13.1	-	*P53*	Regulates endogenous p53 mRNA levels and further induction of p53 protein	HR, NHEJ	53
*BARD1 9'L*	2q35	Oncogene	*BARD1*	Counteracts the effect of miR-203 and miR-101, modulating BARD1 mRNA expression	HR	20
*MALAT1*	11q13.1	Oncogene	*P53*	Inhibits p53 level; possible feedback mechanism by which MALAT1 is also regulated by p53	HR, NHEJ	37, 38
*Linc-ROR*	18q21.31	Tumor suppressor	*P53 translation*	Inhibits the translation of p53 protein that, in turn, promotes the expression of the lncRNA	HR, NHEJ	33, 34
*Evf2 lncRNA*	7q21.3	Oncogene	*BRG1/DLX1*	Inhibits BRG1 ATPase activity and remodeling activity	HR	26

HR: homologous recombination, NHEJ: non-homologous end joining

***BRCA1-associated RING domain protein 1 9'L (BARD1 9'L)***

This lncRNA is transcribed from an alternative intronic promoter of *BARD1* and up-regulates the expression of an oncogenic *BARD1* isoform. *BARD1* has some variants with tumor suppressor functions and participates in a variety of cellular processes such as DNA repair, transcriptional regulation, chromatin remodeling, cell cycle check-point control, and mitosis. Additionally, *BARD1* has a critical role in the preservation of genomic stability (20). BARD1 stabilizes BRCA1 protein by producing a heterodimeric really interesting new gene (RING)-RING complex, and facilitates its role in HR repair. However, the existence of an oncogenic *BARD1 *splice variant in some cancers has been associated with the prevention of proper BRCA1 function. It has been demonstrated that loss of BARD1 function through the expression of the oncogenic splice variant leads to a more invasive phenotype in malignant cells which is accompanied with diminished RAD51 foci establishment and reduced nuclear BRCA 1 protein accumulation (21). Consequently, *BARD1 9'L* is regarded as an oncogenic lncRNA over- expressed in a variety of human tumors (22).

***DNA damage- sensitive RNA1 (DDSR1)***

It is a lncRNA whose expression is triggered following DSB in an ATM- nuclear factor kappa B (NF- κB) pathway-dependent mode. In the absence of *DDSR1* cell proliferation and DDR signaling is defective as well as DNA repair capacity by HR. The latter has been shown by abnormal assembly of BRCA1 and ubiquitin interaction motif containing 1 (UIMC1 or RAP80) at DSB sites. Consistent with its function in regulating HR, *DDSR1* cooperates with BRCA1 and heterogeneous nuclear ribonucleoprotein U like 1 (hnRNPUL1), an RNA-binding protein participating in DNA end excision (23). Its participation in HR pathway has been demonstrated in figure 2. Furthermore, in line with the role of lncRNAs in the regulation of gene expression, RNAi treatment targeted against *DDSR1* has led to differential expression of more than 100 genes implicated in several fundamental pathways (24). These genes can be classified into the groups of cell death and survival (*CDK6*,

**Fig. 2:**
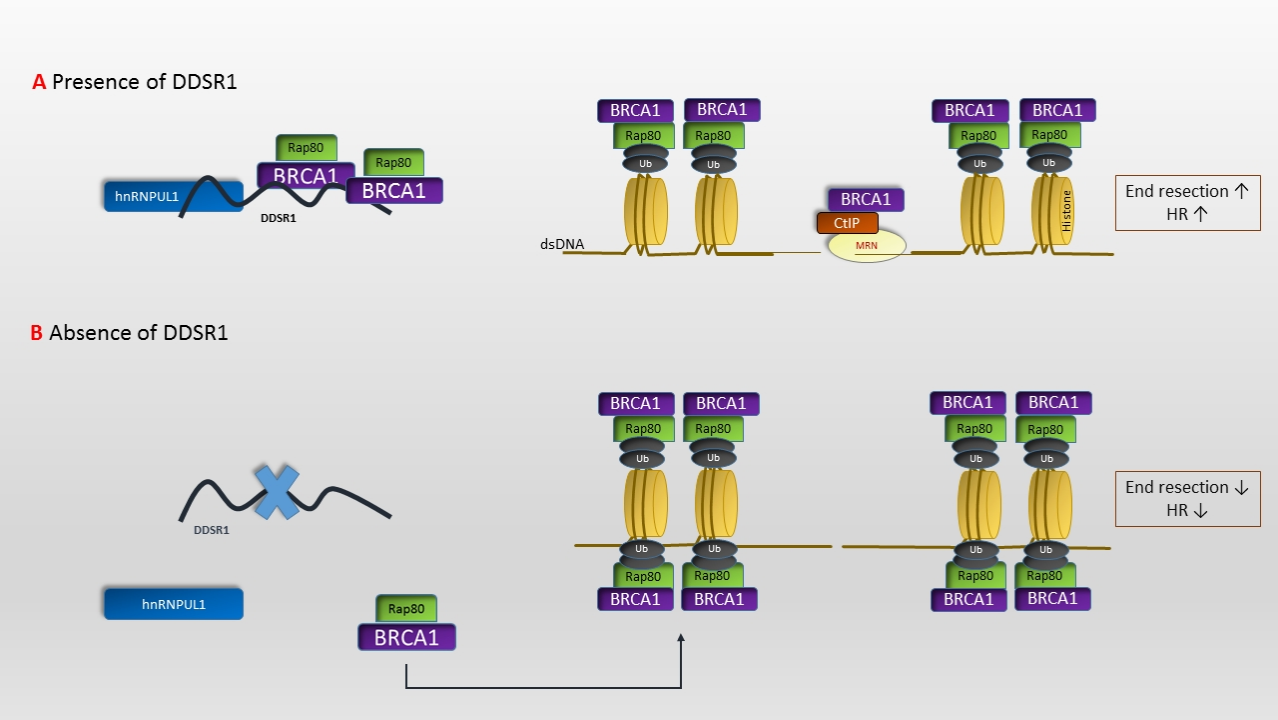
Participation of DNA damage-sensitive RNA1 in homologous recombination. DNA damage-sensitive RNA1* (DDSR1)* is involved in regulation of homologous recombination (HR) pathway via its attachment to breast cancer 1 (BRCA1) and heterogeneous nuclear ribonucleoprotein U like 1 (hnRNPUL1) (2A). In the absence of *DDSR1* (2B) cell proliferation, DNA damage response (DDR) signaling and DNA repair capacity by HR are defective. The latter has been shown by abnormal assembly of BRCA1 and ubiquitin interaction motif containing 1 (UIMC1 or RAP80) at double strand break (DSB) sites. *DDSR1* cooperates with BRCA1 and heterogeneous nuclear ribonucleoprotein U like 1 (hnRNPUL1), an RNA- binding protein participating in DNA end excision. HR is started by a 5′–3′ degradation of one strand at both sides of the break, making stretches of single-stranded DNA (ssDNA) that is then coated by the ssDNA binding protein complex RPA. This process is called DNA-end resection (57). Ub: ubiquitination, CtIP: CtBP-interacting protein, MRN complex: Mre11, Rad50, Nbs1

*E2F7*, *OLR1*, *JAG1*, *TNFSF18*, *MCM6*, *IFIT3*) and DNA replication, recombination, and repair (*CENPW*, *MCM6*, *ANP32E*, *HELLS*). Notably, depletion of *DDSR1* resulted in upregulation of some p53 target genes (*DRAM*, *DHRS3*, *HMOX1*), implying that this lncRNA contributes in transcriptional suppression of p53 target genes (23).

***DLX6 antisense RNA 1 (DLX6- AS1 or Evf 2 lncRNA)***

This lncRNA is the first identified ultra-conserved lncRNA. It has been shown to promote the assembly of transcriptional activators (Distal-less homeobox (DLX) homeodomain proteins) with essential DNA enhancers while suppresses gene expression. Its interaction with Brahma-related gene-1 (BRG1) and DLX1 has a fundamental role in RNA-dependent chromatin remodeling inhibition (25). BRG1 is the catalytic ATPase of the SWItch/Sucrose Non-Fermentable (SWI/SNF) complex that is phosphorylated by ATM and participates in DSB repair (26).

***LncRNA-Jade family plant homeo domain (PHD) finger (LncRNA- JADE)***

This lncRNA has been shown to be up-regulated following DNA damage in an ATM-dependent method but independent of p53. It induces the transcription of Jade1, an important factor in the HBO1 (human acetylase binding to origin recognition complex 1 (ORC1)) histone acetylation complex. Therefore, *lncRNA-JADE* triggers histone H4 acetylation in the DDR. Expression level of this lncRNA has been shown to be elevated in human breast tumors compared with normal breast tissues. Additionally, its knock-down has prevented breast tumor growth *in vivo* which is consistent with its role in breast tumorigenesis. In addition, it has been supposed that *lncRNA-JADE* directly interacts with BRCA1 which leads to recruitment of *lncRNA-JADE* to the p300/CBP coactivator complex. In brief, this oncogenic lncRNA participates in breast carcinogenesis by cooperation with Brca1 and histone acetylation machinery (3).

***Mediator of DNA damage checkpoint 1- antisense RNA (MDC1- AS)***

It is an antisense lncRNA identified by lncRNA microarray analysis in bladder cancer cell lines (27). MDC1 has been known as a vital mediator of the repair of DSB which takes part as an important tumor suppressor via its DNA damage repair function. It is activated following DSB by ATM protein kinase and assembles DDR factors to DSB sites (28). The expression levels of *MDC1-AS* and *MDC1* has been shown to be down-regulated in bladder cancer. Up-regulation of *MDC1-AS* has resulted in increased levels of *MDC1* in these cells. This lncRNA has been demonstrated to exert an inhibitory effect on malignant bladder cancer cells which has been due to up-regulation of *MDC1*. Consequently, this lncRNA may contribute in bladder cancer via up-regulation of its antisense tumor-suppressing gene *MDC1* (27).

***Prostate cancer associated transcript 1 (PCAT1)***

This lncRNA is the first cytoplasmic prostate lncRNA whose expression is associated with treatment response. It has been shown to control cell response to genotoxic stress which has been supported by the converse correlation between *PCAT-1* and *BRCA2* expression in human prostate cancer samples. Its expression leads to a functional insufficiency in HR which is mediated through inhibition of the *BRCA2* tumor suppressor, and subsequently a high sensitivity to small-molecule inhibitors of Poly (ADP-ribose) polymerase 1 (PARP1). Although the exact mechanism of its function is not clarified yet, 5′ portion of the *PCAT-1* RNA, which contains entirely unique sequences, is necessary for the control of *BRCA2* expression (29).

***Transcribed in the opposite direction of RAD51 (TODRA)***

It is transcribed 69 bp upstream to *RAD51*, in the reverse orientation. RAD51 has a critical role in HR and DSB repair and its expression is dysregulated in various cancers leading to genomic instability and tumor progression. The *RAD51* promoter region functions bidirectionally as a strong promoter for both *TODRA* and *RAD51*. This promoter region also comprises an E2F binding site which controls *TODRA* expression, but its effects are opposite to those on *RAD51*. In other words, elimination of the E2F site enhances *RAD51* promoter activity but reduces *TODRA* promoter activity. *TODRA* considerably enhances both the portion of RAD51-positive DNA damage-induced foci and HR repair effectiveness (30).

**LncRNA role in non-homologous end joining**

***LncRNA in non-homologous end joining pathway 1 (LINP1)***

It has been identified as a lncRNA that is up-regulated in human triple-negative breast cancer (TNBC). It has been shown to increase double-strand DNA break repair by providing a scaffold that connects Ku80 and DNA-dependent protein kinase (DNA-PKcs), thus organizing the NHEJ pathway. However, cells deprived of *LINP1* expression can still repair DNA through the NHEJ pathway which suggests that *LINP1 *is not necessary for the NHEJ process. Yet, enforced expression of *LINP1* in such cells can augment NHEJ mediated DNA repair function. *LINP1* expression is regulated by epidermal growth factor receptor (EGFR) activation through the activation of the RAS-Mitogen-activated protein kinase/ERK kinase (MEK)/extracellular-signal-regulated kinase (ERK) pathway and AP1 transcription factors. Besides, the tumor suppressor p53 has been shown to decrease *LINP1* expression via miR-29 by up-regulation of miR-29 that targets *LINP1* RNA. Frequent occurrences of EGFR amplification and TP53 mutations in TNBC have been suggested to up-regulate *LINP1* expression. On the other hand, *LINP1* amplification might augment the response to elevated EGFR activity and loss of TP53 suppression in TNBC. *LINP1* knock-down promoted doxorubicin-induced apoptosis in TNBC cell lines, while overexpression of *LINP1* in an estrogen-receptor positive breast cancer cell line with untraceable *LINP1* inhibited the apoptotic effects of doxorubicin in these cells. In addition, *LINP1* blockade enhances the sensitivity of breast tumor cells to radiotherapy (31).

**LncRNAs with probable roles in both pathways**

***Long intergenic non-protein coding RNA, regulator of reprogramming (Linc-ROR)***

This lncRNA has been shown to contribute in preserving the pluripotency of human embryo stem cells through acting as an “endogenous sponge” to prevent miR-145-dependent differentiation of these cells (32). In addition, it has been suggested to participate in the cancer stem cells properties in tumor bulks (33). Human *lincRNA-ROR* has also been known as a potent negative regulator of p53 which acts through direct interaction with the heterogeneous nuclear ribonucleoprotein I (hnRNP I). A 28-base RoR sequence contains hnRNP I binding motifs and is necessary and adequate for p53 repression. This lncRNA prevents p53-mediated cell cycle arrest and apoptosis. Consequently, RoR-hnRNP I-p53 axis has been suggested as an extra surveillance network for the improved responsiveness of cells to numerous stresses (34). A more recent study has demonstrated the notable lower expression of *lincRNA-ROR* in glioma tissues than in adjacent normal tissues. Knockdown of *lincRNA-ROR* expression has resulted in enhanced cell proliferation, whereas overexpression of *lincRNA-ROR* had the inverse effect. Consequently, the reprogramming-related *lincRNA-ROR* has been suggested as a putative tumor suppressor gene in glioma (35). Although currently no study has demonstrated its exact role in DSB repair, its role in p53 regulation potentiates its effect in the mentioned pathways.

***Metastasis associated lung adenocarcinoma transcript 1 (MALAT1)***

This lncRNA has been shown to be invariably over-expressed in numerous epithelial tumors in which p53 malfunction has been detected (36). Its over-expression has also been correlated with negative outcomes of treatment in patients with osteosarcoma (37). Furthermore, a fragment located at the 3' end of *MALAT1* has a critical function in cell proliferation, migration and invasion in colorectal cancer (38). Considerable *MALAT1* over-expression in the presence of polyoma and papilloma oncoproteins has been shown to disturb or contradict p53 expression/function; so its expression level has been suggested as a biomarker for p53 dysregulation in tumoral tissues (36). Another study has revealed that *MALAT1* modifies the expression of cell cycle genes and is necessary for G1/S transition. Its knock-down has resulted in activation of p53 and its target genes. Besides, *MALAT1*-depleted cells show diminished expre-ssion of B-MYB (MYB proto-oncogene like 2, Mybl2), an oncogenic transcription factor involved in G2/M transition (39). Considering the impor-tance of checkpoint pathways in regulation of DSB repair mechanisms in distinct phases of the cell cycle (40) and the role of p53 in nearly all steps (41), *MALAT1* is anticipated to have important function in DSB repair regulation.

***P21-associated ncRNA DNA damage activated (PANDA)***

It is a 5′-capped and polyadenylated non-spliced lncRNA that is transcribed from *CDKN1A* promoter region in an antisense orientation. Its transcription is up-regulated following DNA damage in a p53-dependent manner. Its main function is the regulation of apoptosis response following DNA damage. In other words, *PANDA* cooperates with the nuclear transcription factor Y subunit alpha (NF-YA) to limit expression of pro-apoptotic genes. Its knock-out significantly sensitized human fibroblasts to apoptosis by doxorubicin (42).

***Telomeric repeat-containing RNA (TERRA)***

Telomeres are transcribed into a lncRNA comprising UUAGGG repeats which is called as *TERRA*. This RNA polymerase II (RNAPII) transcript is present in all eukaryotes (43). Telomeres are special constructions at the end of chromosomes which guard these sites from being identified as sites of DNA damage. As a consequence of telomere shortening or telomere uncapping which is triggered by telomeric repeat-binding factor 2 (TRF2) deficiencies, telomeres provoke a DDR resulting in cellular senescence. It has been demonstrated that TRF2 depletion will lead to up-regulation of *TERRA* and recruitment of lysine demethylase 1A (LSD1) to telomeres (44). It has been also shown that TRF2-deficient telomeres go through telomere fusion processes by NHEJ (45), while TRF2 is suggested to inhibit T-loop HR by suppression of branch migration and/or strand cleavage (46). At uncapped telomeres, LSD1 assembles with meiotic recombination 11 (MRE11), one of the nucleases involved in the processing of 3′ telomeric G overhangs. Conse-quently, *TERRA* interacts with LSD1 to control MRE11 function in the processing of uncapped telomeres (44). 3′ G overhang elimination is a prerequisite for telomere end-joining process by the NHEJ machinery (47). In brief, *TERRA* participates in control of telomerase activity and hetero-chromatin formation at telomeres and contributes in the DDR activated by dysfunctional telomeres. In addition, *TERRA* transcripts have been demons-trated to produce DNA-RNA hybrids at telomeres which can enhance HR between telomeres, post-poning cellular senescence and maintaining genome instability (48).

***Tumor protein p53 pathway corepressor 1 (TRP53COR1, LincRNA-P21)***

This lncRNA has been found to participate in DNA damage and cell cycle control in an attempt to detect non coding transcripts expressed in a p53-dependent manner (49). In the mentioned study, *lincRNA-p21* has been shown to act as a repressor in p53-dependent transcriptional responses. Its knock-down changes the expression of several gene targets which are normally inhibited by p53 (49). It is also necessary for induction of p53-dependent apoptosis resulting from DNA damage by binding to heterogeneous nuclear ribonucleoprotein K (hnRNP-K) (50). Considering the modulatory function of p53 in DNA repair and recombination (51), *lincRNA-p21* is anticipated to play critical role in both HR and NHEJ pathways.

***WD repeat containing antisense to TP53 (WRA-P53)***

It is a natural antisense transcript of p53 that controls *p53* mRNA levels by targeting the 5' untranslated region of *p53* mRNA. Its knock-down has led to notable decrease in *p53 *mRNA and inhibition of *p53* induction following DNA damage. On the contrary, its overexpression has enhanced *p53* mRNA and protein levels and sensitized cells for p53-dependent apoptosis. Its role in regulation of *p53 *has been shown to be exerted via formation of *Wrap53/p53* RNA hybrids (52). Considering the role of p53 in regulation of DSB repairs via both HR and NHEJ, and its function as an important hub between upstream DNA damage signals and subsequent cellular responses such as repair, recombination, and apoptosis (51) its natural antisense is expected to participate in both main DNA repair mechanisms as well.

## Discussion

Several lncRNAs have been shown to participate in regulation of certain steps of DNA repair process including recognition of the DNA damage, signal relays and initiation of the repair. Although the contribution of these lncRNAs has been identified by means of several strategies, microarray technology has been suggested as a valuable method for determination of their role in the DNA damage repair pathway (53). According to data presented above, lncRNAs function has been documented in the regulation of both HR and NHEJ pathways. Some of them have been implicated in modulation of repair pathway choices as well as regulation of gene expression (54). Not surprisingly, they have been shown to be abnormally expressed in cadmium-treated cells and presented as an indication for DNA damage and repair associated with the epigenetic mechanisms (55). Besides, based on the speculation that intergenic regions are frequently transcribed into lncRNAs, lncRNAs may be implicated in processing of short RNAs which are essential for DNA repair (17). This cooperation of lncRNAs with miRNAs in regulation of gene expression should be assessed in experimental models of DNA repair. LncRNA has been shown to be involved in tumourigenesis of many cancers as well. On the other hand, defects in DNA repair system are associated with cancer. While mutations in DSB repair components are frequently detected in hereditary cancers, impaired DSB repair in sporadic cancers might be explained by altered regulatory mechanisms such as lncRNAs (29). LncRNAs role in the regulation of DSB repair system is exerted epigenetically through chromatin-modifying complexes as well as posttranscriptional control of their target genes (29). Assessment of lncRNAs involved in DNA repair system would pave the way for identification of their role in cancer development and drug response considering the significance of DDR in determination of response to radiotherapy and chemotherapy (31). *LINP1* provides a significant example of this hypothesis as its overexpression has been shown to increase resistance to genotoxic offenses and its down-regulation enhances chromosome instability. These data have led to suggest the application of miR-29 mimetics for *LINP1* targeting as a beneficial strategy for therapeutic prevention of NHEJ in definite clinical situations (56). Future studies are needed to explore the physiologic role of lncRNAs in DNA repair pathways as well as the functional consequences of their dysregulation in human disorders including cancer.

## Conflict of interest

The authors declared no conflict of interest. 
